# Discovery and Mechanistic Elucidation of Glycyrrhizic Acid Composite Gel in Promoting Wound Healing: A Modernized Study Based on Shengji Yuhong Ointment

**DOI:** 10.3390/ph18111737

**Published:** 2025-11-14

**Authors:** Hai-Xin Liu, Min-Yu Wang, Ying-Wei Li, Bin Xu, Zi-Xuan Wang, Xiang-Long Meng, Hui-Fang Li, Shi-Yuan Wen

**Affiliations:** 1 College of Traditional Chinese Medicine and Food Engineering, Shanxi University of Chinese Medicine, Taiyuan 030606, China; 2 College of Basic Medical Sciences, Shanxi Medical University, Taiyuan 030606, China

**Keywords:** wound, glycyrrhizic acid, PPIA, gel, Shengji Yuhong Ointment

## Abstract

**Objectives**: Shengji Yuhong Ointment (SJYHO) is a classic Traditional Chinese Medicine prescription used for refractory wounds, yet its systemic pharmacological mechanisms remain unclear. This study aimed to identify its key active compounds and develop a simplified, effective topical formulation. **Methods**: We employed an integrated approach, combining network pharmacology and machine learning to screen the key constituents and core targets of SJYHO. The lead compound, glycyrrhizic acid, was formulated into a hydrogel (GA-Gel). Its therapeutic efficacy was evaluated in a full-thickness excisional wound model in Sprague-Dawley rats over 21 days, assessing healing kinetics, histology, and pain behavior. The interaction between glycyrrhizic acid and the identified target PPIA, along with its immunomodulatory effects, was validated through molecular docking, molecular dynamics simulation, and RT-qPCR. **Results**: Our integrated analysis identified PPIA as the core target and glycyrrhizic acid as a key bioactive component of SJYHO. Animal experiments demonstrated that GA-Gel significantly accelerated wound closure, which was driven by its multi-faceted actions: reducing inflammation, promoting collagen deposition, alleviating pain, and modulating late-stage angiogenesis. Mechanistically, we confirmed that glycyrrhizic acid stably binds to PPIA. Furthermore, GA-Gel treatment mediated wound immune infiltration by specifically regulating CD8^+^ T cells, neutrophils, and memory B cells, an effect that was dependent on PPIA targeting. **Conclusions**: This study demonstrates that glycyrrhizic acid, formulated as GA-Gel, recapitulates the wound-healing benefits of SJYHO by specifically targeting PPIA and modulating the immune microenvironment. Our findings not only elucidate a key mechanistic pathway but also present GA-Gel as a rationally designed, clinically translatable therapy for acute and chronic wounds.

## 1. Introduction

Wound, a common skin injury, has a complex healing process involving hemostasis, inflammation, proliferation, and remodeling [1]. During the proliferative phase, angiogenesis, driven by factors like VEGF and FGF from inflammatory cells and fibroblasts, is crucial. These factors stimulate new capillary growth, supplying the wound with oxygen, nutrients, and immune cells [2]. The inflammatory phase involves platelets, macrophages, and neutrophils releasing pro-inflammatory cytokines to clear pathogens and debris [3]. Collagen remodeling provides a scaffold for cell migration and proliferation. Its degradation products act as chemotactic signals for macrophages, modulating inflammation and angiogenesis [4]. In the remodeling phase, type III collagen is gradually replaced by type I collagen to strengthen the wound [5]. These interconnected processes collectively determine the efficiency and quality of wound healing.

Shengji Yuhong Ointment (SJYHO), a traditional Chinese medicine, promotes wound healing by detoxifying, reducing swelling, forming new tissue, and alleviating pain [6]. It stimulates granulation tissue growth and improves microcirculation, providing essential blood supply for healing. Its components, such as *Lithospermum erythrorhizon* Siebold & Zucc. and *Glycyrrhiza uralensis* Fisch., have antibacterial and anti-inflammatory properties that curb bacterial growth, reduce inflammation, lower infection risk, and enhance immune function by activating lymphocytes [7,8]. The ointment also regulates growth factors like bFGF, FGF, EGF, PDGF, TGF-β, and VEGF. In the early wound healing phase, it promotes angiogenesis and fibroblast proliferation. During the remodeling phase, it reduces bFGF levels to promote collagen degradation and inhibits angiogenesis to minimize scarring [9,10]. Additionally, it inhibits pro-inflammatory cytokines such as TNF-α, IL-1, and IL-6 to create a favorable healing environment [11]. By facilitating epithelial cell growth and collagen synthesis, SJYHO accelerates wound repair and strengthens the wound [9]. However, the specific targets and mechanism by which SJYHO promotes wound healing have not yet been reported.

Glycyrrhizic acid, a pentacyclic triterpenoid from glycyrrhiza, has broad pharmacological effects, including anti-inflammation, anti-oxidation, anti-tumorigenesis, and immune regulation [12]. It inhibits COX and LOX, reducing prostaglandin and leukotriene production, and suppresses the NF-κB pathway to lower pro-inflammatory cytokine expression, thus easing inflammation and creating a favorable environment for wound healing [13,14]. Glycyrrhizic acid modulates the immune response by enhancing macrophage phagocytosis, promoting lymphocyte proliferation, and balancing Th1/Th2 cells, thereby bolstering the body’s immune defense and aiding wound repair [15,16]. It also combats oxidative stress, further supporting tissue recovery [17]. Glycyrrhizic acid is formulated into various products, including injectables (e.g., diammonium glycyrrhizate and compound glycyrrhizin injections), orals (e.g., diammonium glycyrrhizate capsules and compound glycyrrhizin tablets), topicals (e.g., glycyrrhizic acid gels), and glycyrrhizic acid tablets [18]. Potassium glycyrrhizate and other glycyrrhizic acid-based drugs promote granulation tissue formation and fibroblast proliferation. They also speed up re-epithelialization and angiogenesis, collectively accelerating wound healing [19].

All kinds of trauma can cause wounds and trigger an immune response to repair the damage. Without timely treatment, serious physical complications may occur [20]. SJYHO is used to promote wound healing. However, its use requires caution due to potential allergy risks and possible long-term toxicity. Previous studies have primarily described the phenotypic effects of SJYHO, but a systems-level understanding of its key bioactive ingredients and their specific cellular targets is critically needed. To address this, our study employed an integrated strategy combining network pharmacology and machine learning to systematically identify the core active compound and its primary target from the complex mixture of SJYHO. We further sought to translate this discovery into a simplified, target-specific hydrogel formulation. This study explores its therapeutic mechanisms to develop new wound-healing drugs. Based on these mechanisms, the study finds and reveals how glycyrrhizic acid-based composite gels (GA-Gel) aid wound repair.

## 2. Results

### 2.1. Target Identification for SJYHO in Wound Healing

**Figure 1:**
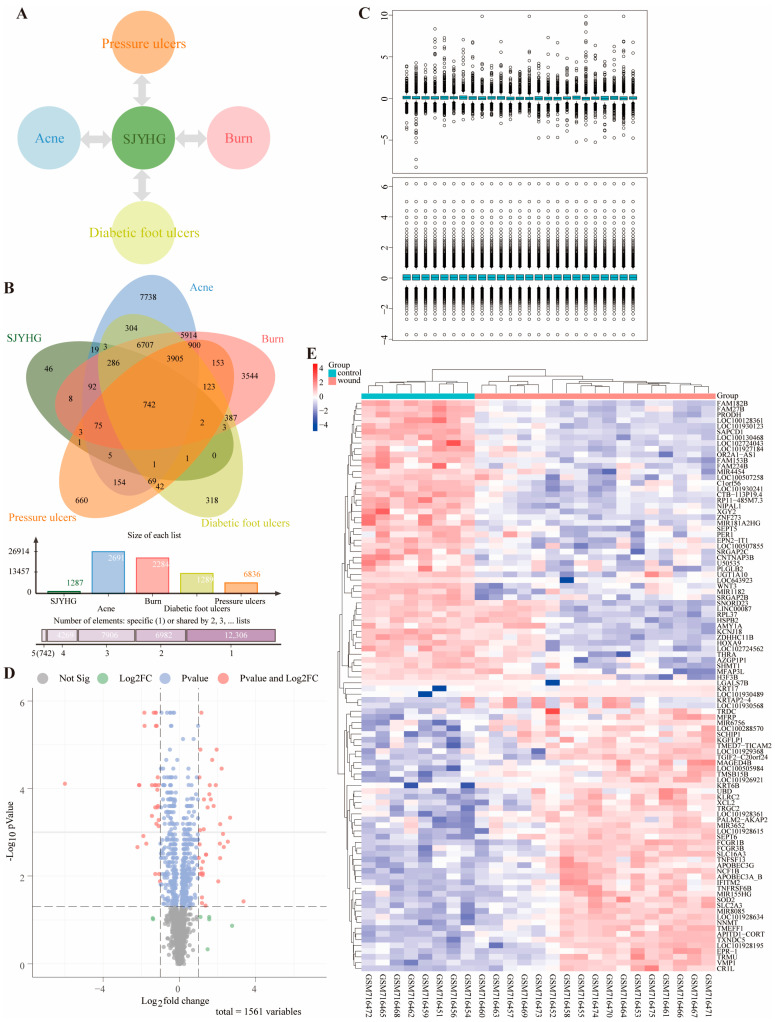
Target identification for Shengji Yuhong Ointment (SJYHO) in wound healing. (**A**) SJYHO is clinically indicated for the treatment of wounds such as acne, burns, diabetic foot ulcers, and pressure ulcers. (**B**) Venn analysis of SJYHO targets intersected with targets of the four traumatic diseases. (**C**) Normalization analysis of the trauma-related GSE28914 dataset. (**D**) Volcano plot illustrating differential gene expression in the GSE28914 dataset. (**E**) Heat map of the top 50 and bottom 50 representative genes; the color scale indicates expression levels, with red denoting high expression and blue denoting low expression.

SJYHO is clinically used to heal wounds like acne, burns, diabetic foot ulcers, and pressure ulcers. TCMIP database analysis shows SJYHO has 137 active components. After analyzing these in TCMIP, SwissTargetPrediction, and PharmMapper databases, 1287 potential targets were found. Using GeneCards and CTD databases, we obtained disease-related targets: 26,914 for acne, 22,844 for burns, 12,893 for diabetic foot ulcers, and 6836 for pressure ulcers. Figure 1A shows the link between SJYHO and these diseases. The intersection of SJYHO and the four diseases yielded 742 potential targets for wound healing (Figure 1B). Data from the GPL570 platform’s GSE28914 dataset were standardized (Figure 1C). The analysis found 387 DEGs, including 174 up-regulated and 213 down-regulated genes. Figure 1D presents a volcano plot of the DEGs. The heatmap displays expression differences of the top and bottom 50 genes between normal and wounded samples (Figure 1E).

### 2.2. PPIA and Glycyrrhizic Acid Are Likely the Key Factors in SJYHO for Wound Healing

**Figure 2:**
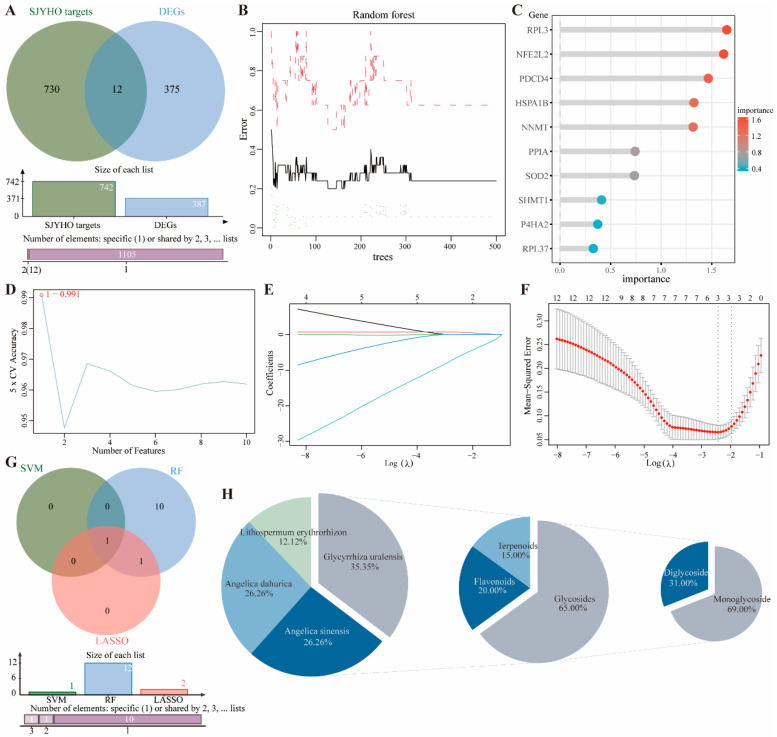
PPIA and glycyrrhizic acid are likely the key factors in SJYHO for wound healing. (**A**) Venn analysis of the potential targets of SJYHO and the DEGs from the GSE28914 dataset. (**B**) The RF analysis. (**C**) A scatter plot showed genes’ importance. (**D**) The SVM accuracy plot. (**E**) The regression path plot of LASSO analysis. (**F**) The cross-validation plot of LASSO analysis. (**G**) Venn analysis of the three machine learning algorithms. (**H**) PPIA was traced back to its source compounds and classified in three hierarchical tiers. Level 1 grouped the compounds by raw-herb categories; Level 2 focused on licorice and further subdivided them by chemical class; Level 3 drilled down into the glycoside subset. A multi-layer pie chart was generated to visualize these data, with distinct colors representing each category.

A Venn analysis of the potential targets of SJYHO and the DEGs from the GSE28914 dataset found 12 common genes, SOD2, MTHFD1, PPIA, SHMT1, HSPA1B, CHRNA7, P4HA2, NNMT, PDCD4, NFE2L2, RPL3, and RPL37 (Figure 2A). Machine learning was performed on these 12 genes. The RF analysis showed the OOB error stabilized at around 310 trees (Figure 2B), and a scatter plot showed the genes’ importance (Figure 2C). The SVM accuracy plot indicated an accuracy of 99.1% at a feature number of 1 (Figure 2D). LASSO analysis followed. The regression path plot suggested two variables with a coefficient of 0 (Figure 2E), and the cross-validation plot showed three variables could be basically determined (Figure 2F).

A Venn analysis of the three machine learning algorithms found PPIA as the unique core gene (Figure 2G). Tracing back from the PPIA protein to its components and classifying them by TCM categories found Glycyrrhiza with 20 components, Angelica with 15, Paeonia with 15, and Lithospermum with 7. Further classification by compound types found Glycyrrhiza had thirteen glycosides, four flavonoids, and three terpenoids. A third-level classification of glycosides found nine monosaccharides and four disaccharides. A composite pie chart visualized the data with colors representing different categories (Figure 2H). Since monosaccharides, especially glycyrrhizic acid, were the most abundant and unique components in Glycyrrhiza, glycyrrhizic acid was chosen as the main subject for the experiment.

### 2.3. Glycyrrhizic Acid Composite Gel (GA-Gel) Promotes Wound Healing

**Figure 3:**
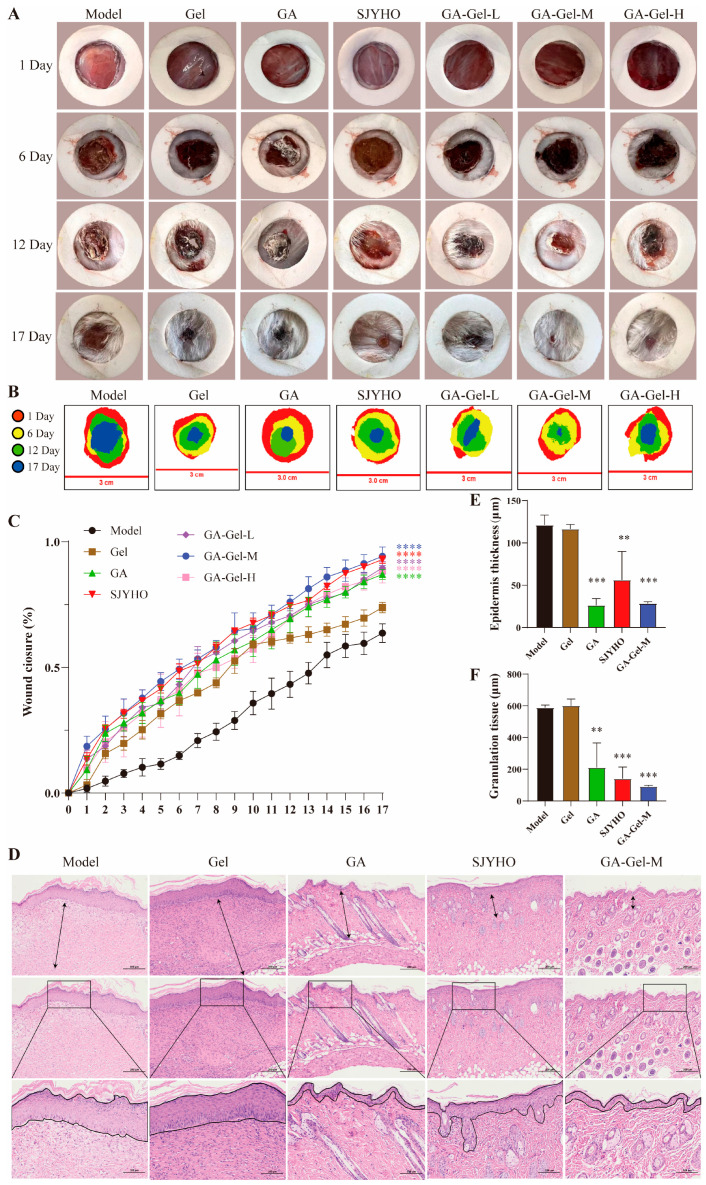
Glycyrrhizic acid-containing composite gel (GA-Gel) promotes wound healing. (**A**) The wound-healing progress on days 1, 6, 12, and 17. (**B**) The healing process of the full-thickness skin injury in mice. (**C**) 17-day wound closure curve. (**D**) H&E staining after 17 days of treatment. The black arrow represents the granulation tissue and the solid black line is a magnified view of the selected area of the rectangle, representing the new epidermis (**E**) Wound epidermis thickness. (**F**) Granulation tissue thickness, mainly including glands in the dermis. Data shown are the mean ± SEM of three independent experiments. ** *p* < 0.01, *** *p* < 0.001, **** *p* < 0.0001, compared with the control group.

To verify the potential of GA-Gel in wound repair, this study used a mouse full-thickness injury model. Figure 3A,B show the wound-healing progress on days 1, 6, 12, and 17, and illustrate the healing process of the full-thickness skin injury in mice. Results indicate that among all groups, the GA-Gel-M group exhibited the fastest wound healing over the 17-day period, with other groups ranking as SJYHO, GA-Gel-L, GA-Gel-H, and GA, all showing significantly faster healing than the Control group (*p* < 0.0001, Figure 3C). Notably, the GA-Gel-M group showed no inflammation, redness, or pus formation throughout the experiment, unlike the Control group, which experienced redness during recovery. This suggests that GA-Gel effectively promotes wound healing.

After 17 days of treatment, H&E staining showed remarkable pathological changes in the skin tissue at the wound site (Figure 3D). Compared to the Control group, the GA-Gel-M group demonstrated an intact epidermal structure, a significant reduction in wound thickness (*p* < 0.001, Figure 3E), and evenly distributed glands in the dermis (*p* < 0.001, Figure 3F), indicating the most effective healing. The SJYHO group also exhibited an intact epidermal structure (*p* < 0.01, Figure 3E), reduced wound thickness, and the presence of dermal glands (*p* < 0.001, Figure 3F). Similarly, the GA group showed a mostly intact epidermal structure (*p* < 0.001, Figure 3E) with minor hyperplasia and some new hair follicles in the dermis (*p* < 0.01, Figure 3F). In contrast, the Gel group had minimal epidermal repair, no hair follicle regeneration, and no significant improvement (Figure 3E,F). These results confirm that GA-Gel effectively promotes hair follicle regeneration and skin tissue reconstruction in mouse wounds.

### 2.4. GA-Gel Relieves Pain and Increases Collagen Deposition

**Figure 4:**
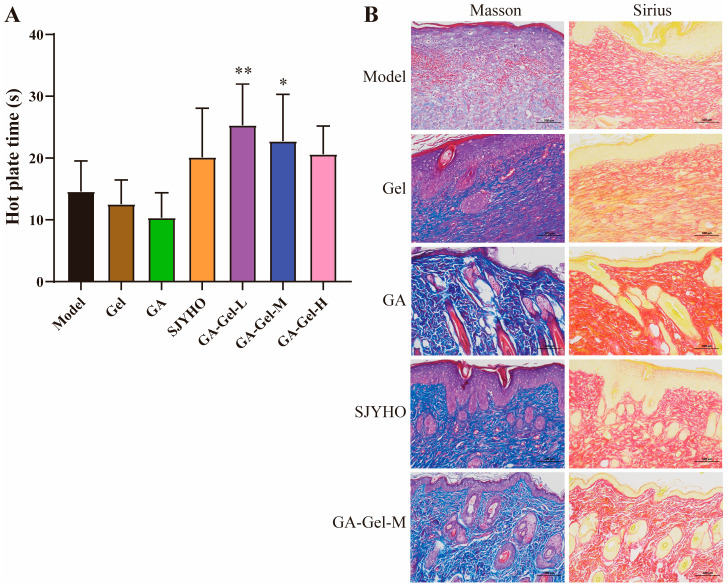
GA-Gel relieves pain and increases collagen deposition. (**A**) The pain thresholds between different groups. (**B**) The representative images of Masson’s trichrome (**left**) and Sirius Red (**right**) staining of wounds. Data shown are the mean ± SEM of three independent experiments. * *p* < 0.05, ** *p* < 0.01, compared with the control group.

To determine whether GA-Gel has analgesic effects in wounds, we compared pain thresholds between different groups. Compared with the model group, the SJYHO group and low-, medium-, and high-dose GA-Gel groups all increased pain thresholds. Notably, the low-dose and medium-dose GA-Gel groups showed significant increases (*p* < 0.05, Figure 4A), indicating a strong analgesic effect. However, the GA group had the shortest duration of effect, possibly due to its poor water-solubility, which may limit its therapeutic efficacy.

Collagen is a crucial component of skin tissue and plays a key role in wound healing. Therefore, we assessed the impact of GA-Gel on collagen deposition in wounds. Figure 4B shows representative images of Masson’s trichrome (left) and Sirius Red (right) staining of wounds. After 17 days of treatment, the GA, SJYHO, and GA-Gel-M groups exhibited significantly higher collagen deposition compared with the Control group (Figure 4B). The microscopic structure of collagen in these groups showed a uniform network, similar to normal skin. In contrast, the Control and Gel groups had collagen fibers arranged in anisotropic bundles, indicative of scar tissue. These results demonstrate that GA-Gel effectively promotes wound healing and provides an optimal environment for scar-free wound repair.

### 2.5. GA-Gel Reduces Inflammation and Late-Stage Angiogenesis

**Figure 5:**
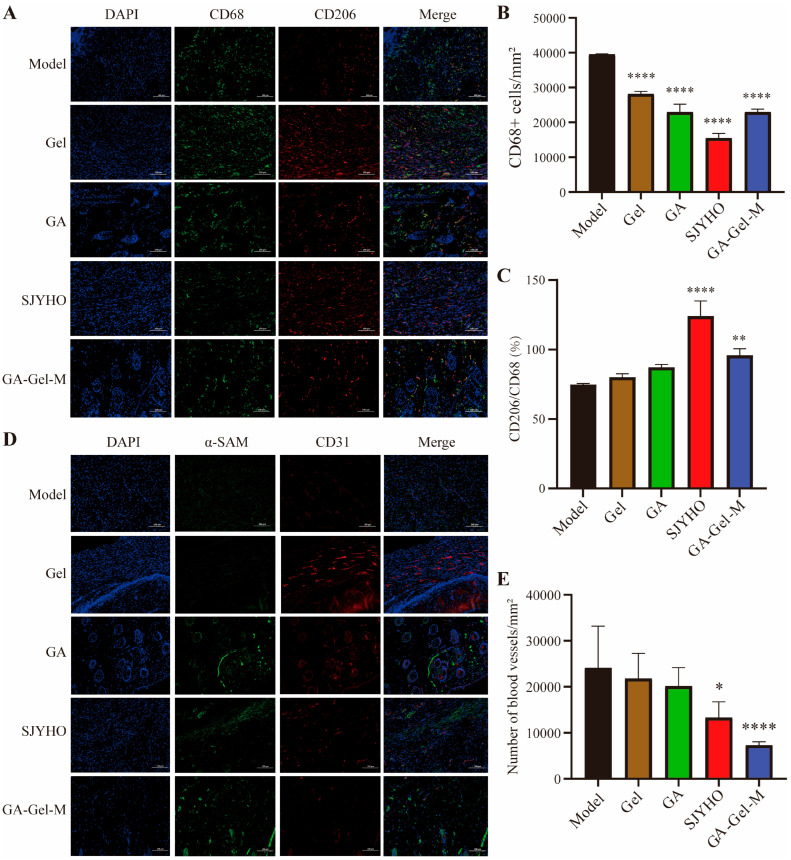
GA-Gel reduces inflammation and late-stage angiogenesis. (**A**) The dual-staining for CD68 and CD206 after 17 days of GA-Gel treatment. (**B**) The CD68-positive cells among different groups. (**C**) The CD206/CD68-positive cells among different groups. (**D**) Immunofluorescence staining for CD31 and α-SMA. (**E**) Vascular density. Data are represented as mean ± SEM. * *p* < 0.05, ** *p* < 0.01, **** *p* < 0.0001, compared with the control group.

Inflammation and angiogenesis are crucial processes in wound healing. Therefore, we assessed the effects of GA-Gel on these processes. CD68 is a marker for mouse macrophages, while CD206 is a marker for anti-inflammatory M2 macrophages. After 17 days of GA-Gel treatment, we performed dual-staining for CD68 and CD206 (Figure 5A). Compared with the Control group, the CD68-positive cells of other groups were significantly decreased (*p* < 0.05, Figure 5B), while both the SJYHO and GA-Gel-M groups showed a significant increase in CD206/CD68-positive cells (*p* < 0.05, Figure 5C), indicating strong anti-inflammatory effects of GA-Gel.

Early-stage wound healing relies on robust angiogenesis to restore perfusion, whereas late-stage remodeling actively suppresses neovascularization to prevent hypertrophic scarring. CD31 is a major marker for vascular endothelial cells, and α-SMA (smooth muscle actin) is a marker for smooth muscle cells, which are widely used to evaluate angiogenesis. After 17 days of GA-Gel treatment, we performed immunofluorescence staining for CD31 and α-SMA (Figure 5D). The GA-Gel-M group showed a significant decrease in vascular density compared with the Control group (*p* < 0.05, Figure 5E). Overall, these data demonstrate that GA-Gel suppresses inflammation and late-stage angiogenesis during wound healing, which is beneficial for scar-free wound repair.

### 2.6. PPIA May Mediate Immune Infiltration of Wound

**Figure 6:**
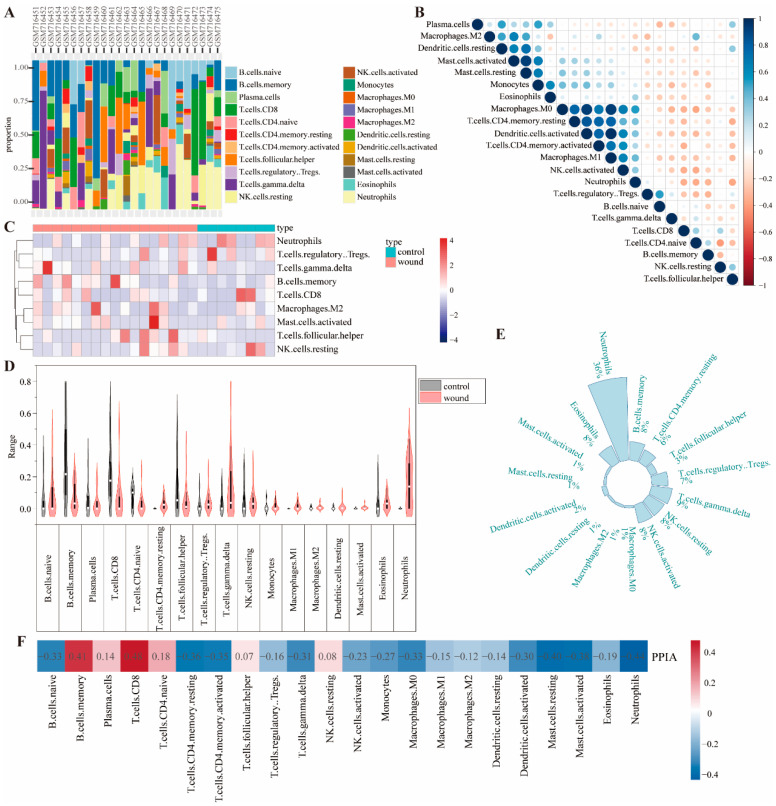
PPIA may mediate immune infiltration of wound. (**A**) Immune cell abundance. (**B**) Correlations between different immune cells in wound. (**C**) A heatmap of immune cells in various samples. (**D**) Violin plots of immune cell expression differences between Wound and Control groups. (**E**) CIBERSORT analysis of immune infiltration in wound samples. (**F**) The correlation between PPIA gene and immune cell. Red indicates a positive correlation. The darker the red color, the stronger the correlation. Blue represents a negative correlation. The darker the blue color, the stronger the correlation.

The inflammatory phase is a critical component of wound healing, and as such, the immune infiltration in wounds was analyzed. Immune cell abundance across samples is shown in Figure 6A. Correlations between different immune cells in wound are presented in Figure 6B. A heatmap displays relative levels of immune cells in various samples (Figure 6C). Violin plots revealed significant immune cell expression differences between Wound and Control groups, mainly in memory B cells, neutrophils, and CD8^+^ T cells (Figure 6D). CIBERSORT analysis indicated immune infiltration in wound samples, with neutrophils accounting for 36% (Figure 6E). The PPIA gene exhibited significant heterogeneity in immune cell infiltration. It showed the strongest correlation with CD8^+^ T cells (positive), followed by neutrophils (negative) and memory B cells (positive) (Figure 6F). Thus, PPIA likely mediates wound immune infiltration by regulating CD8^+^ T cells, neutrophils, and memory B cells.

### 2.7. GA-Gel Promotes Wound Healing by Targeting PPIA

**Figure 7:**
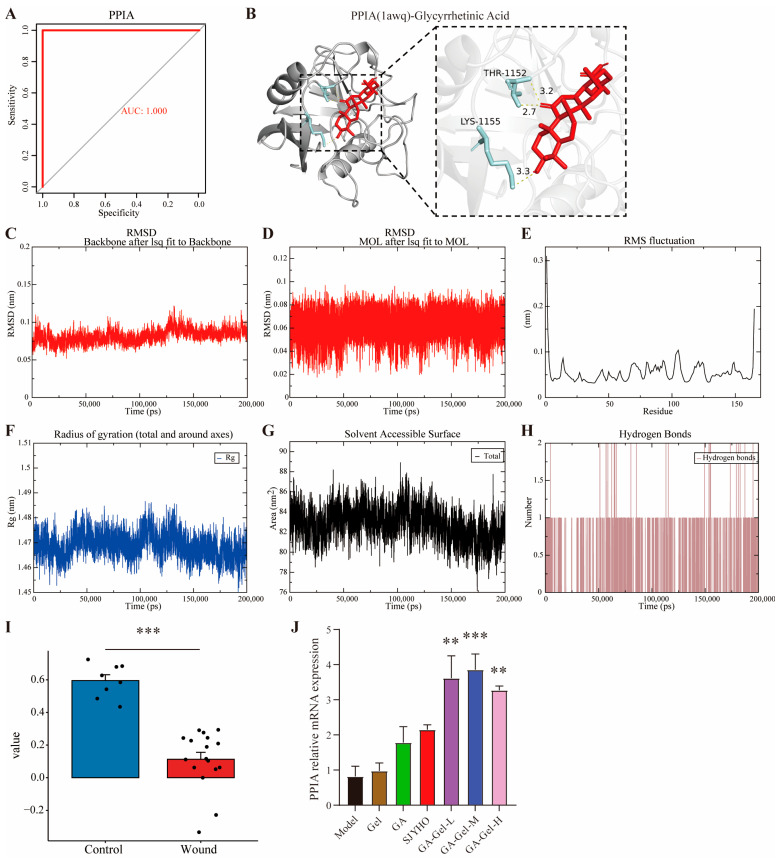
GA-Gel promotes wound healing by targeting PPIA. (**A**) ROC analysis of PPIA. (**B**) Molecular docking experiments between PPIA and glycyrrhetinic acid. (**C**) The RMSD values of the protein backbone. (**D**) The ligand stability of molecular dynamic simulations. (**E**) The RMSF values of residue. (**F**) The Rg values of molecular dynamic simulations. (**G**) SASA. (**H**) Hydrogen bond numbers. (**I**) The PPIA expression from the GSE28914 dataset. (**J**) The mRNA expression level of PPIA by RT-qPCR. Data are represented as mean ± SEM. ** *p* < 0.01, *** *p* < 0.001, compared with the control group.

We analyzed whether glycyrrhizic acid promotes wound healing by targeting PPIA. The ROC analysis of PPIA showed an AUC of 1.0, indicating PPIA’s potential as a key biomarker in wounds (Figure 7A). Molecular docking experiments between PPIA and glycyrrhetinic acid were performed 10 times. Lower binding energy indicates a more stable ligand–receptor binding conformation and a higher likelihood of interaction. The minimum binding energy was −6.9 kcal/mol, suggesting a moderate-strength interaction. Visualization revealed that PPIA is linked to two residues of Glycyrrhetinic Acid through three hydrogen bonds (Figure 7B). During the remodeling of PPIA by Alphafold, a pTM score above 0.5 means the overall predicted fold for the complex might be similar to the true structure. With a resulting pTM score of 0.96, it indicates that the modeling outcome closely resembles the actual structure. In molecular dynamic simulations, the RMSD values of the protein backbone (Figure 7C) and ligand stabilized (Figure 7D), indicating a stable PPIA-glycyrrhetinic acid complex. Most residues had low RMSF values, reflecting protein stability, while higher values indicated flexible regions (Figure 7E). The Rg values declined and stabilized after 50 ns, showing a more compact protein structure (Figure 7F). SASA decreased, implying stable solvation (Figure 7G). Hydrogen bond numbers stabilized after 50 ns, confirming stable interactions (Figure 7H). These data indicate that after 50 ns of simulation, the PPIA-glycyrrhetinic acid system reached a stable state, with the ligand remaining stably bound at the binding site without significant displacement. These data show stable PPIA-glycyrrhetinic acid binding with potential biological activity. The PPIA expression from the GSE28914 dataset was decreased in the wound group compared to the control group (*p* < 0.001, Figure 7I). Importantly, RT-qPCR reveals that the mRNA expression level of PPIA was increased after GA-Gel treatment (*p* < 0.05, Figure 7J). The results above prove that GA-Gel promotes wound healing by targeting PPIA.

## 3. Discussion

SJYHO is used clinically for wounds like acne, burns, diabetic foot ulcers, and pressure ulcers. Network pharmacology suggests PPIA and glycyrrhizic acid are key for its healing effects. When formulated into a composite gel (GA-Gel), animal studies confirm it accelerates wound healing by reducing inflammation, inhibiting late-stage angiogenesis, alleviating pain, and enhancing collagen deposition. PPIA likely mediates wound immune infiltration by regulating CD8^+^ T cells, neutrophils, and memory B cells. Importantly, GA-Gel achieves these effects by targeting PPIA.

Glycyrrhizic acid has been shown to significantly enhance wound healing. They exhibit potent anti-inflammatory and antibacterial activities [21], which help in reducing inflammation and preventing infections at the wound site. Glycyrrhizic acid can form hydrogels that create a moist environment conducive to efficient wound healing. These hydrogels, when combined with other materials like carboxymethyl chitosan, not only improve the mechanical properties and stability of the wound dressing but also promote angiogenesis and tissue regeneration [22]. Furthermore, glycyrrhizic acid has been found to suppress wound inflammation, promote the formation of new granulation tissue, and stimulate tissue re-epithelialization [23]. It also aids in the absorption of wound exudates and accelerates the healing process by promoting cell proliferation and migration [24]. Overall, glycyrrhizic acid has shown great potential in promoting wound healing and tissue regeneration. Glycyrrhizic acid is used in medicine and as a natural sweetener and preservative in foods like candies, beverages, and ice creams. Its anti-inflammatory and antioxidant properties also make it useful in cosmetics for skin-whitening, freckle-removing, and anti-allergy products. Overall, glycyrrhizic acid has broad applications and promising prospects across these fields.

Immune cells play an important role in wound healing. Neutrophils are the first to arrive, clearing pathogens and debris via phagocytosis and releasing enzymes/ROS. Their activity peaks early and declines as healing progresses [25]. Macrophages transition from pro-inflammatory (M1) to anti-inflammatory (M2) phenotypes [26]. CD4^+^ T cells (helper T cells) modulate the immune response by differentiating into subsets, which help activate B cells and macrophages [27]. CD8^+^ T cells (cytotoxic T cells) eliminate infected or damaged cells [28]. In summary, immune cells act at different stages of wound healing to ensure efficient tissue repair. Their balanced activity is crucial for proper wound healing, and imbalance can lead to impaired healing or chronic inflammation. Studies show that PPIA expression is negatively correlated with immune checkpoint levels in B cells, CD4^+^ T cells, CD8^+^ T cells, macrophages, and dendritic cells, suggesting a role for PPIA in modulating these cells’ functions [29]. Our research found that neutrophils are the most prevalent immune cells in wound sites, followed by T cells, and that PPIA exhibits strong correlations with CD8^+^ T cells and neutrophils during wound healing.

Glycyrrhizic acid modulates immune cells to reduce inflammation and promote tissue repair [23]. It inhibits neutrophil infiltration and activation by suppressing inflammatory mediators, reducing tissue damage. In macrophages, it downregulates pro-inflammatory cytokines (e.g., TNF-α, IL-6) and upregulates anti-inflammatory cytokines (e.g., IL-10), enhancing phagocytic activity and promoting the M2 phenotype for tissue repair. In T lymphocytes, it suppresses pro-inflammatory cytokines (e.g., IL-2, IFN-γ), promotes regulatory T-cell differentiation, and enhances inhibitory receptor expression, preventing excessive immune responses and tissue damage. Overall, glycyrrhizic acid balances immune responses to facilitate wound healing. Glycyrrhizic acid has anti-inflammatory and immunomodulatory effects and may enhance its efficacy by interacting with PPIA. It might target the PPIA protein to regulate CD8^+^ T cells and neutrophils, mediating the inflammatory phase of wound healing.

Wound healing begins as fibroblasts migrate to the wound site, producing collagen and other extracellular matrix components. Angiogenesis supplies the wound with oxygen and nutrients. As collagen and the matrix remodel, the wound’s structure and function gradually restore, with tensile strength increasing over time. PPIA protein may influence extracellular matrix remodeling by affecting collagen folding [29]. Our study shows that glycyrrhizic acid affects collagen remodeling and inhibits late-stage wound angiogenesis, likely by targeting PPIA protein, thus accelerating scar-free wound healing. Gel formulations offer several benefits for wound healing [30], so we prepared glycyrrhizic acid into a compound gel, which is beneficial for wound healing.

While our study confirmed the pivotal involvement of PPIA, its role as a multifunctional signaling hub suggests it orchestrates healing by modulating key pathways. Based on established literature, we propose that PPIA likely mediates GA-Gel’s effects through coordinated regulation of the NF-κB, MAPK, and TGF-β pathways. Firstly, the accelerated inflammation resolution observed in our model can be plausibly explained by PPIA’s documented interaction with the NF-κB pathway. Extracellular PPIA is known to activate NF-κB signaling via its receptor CD147 [31,32]. However, the precise temporal modulation of this axis is crucial. We hypothesize that GA-Gel-induced PPIA may contribute to a negative feedback mechanism that fine-tunes and ultimately dampens NF-κB activation, thereby preventing excessive inflammation and facilitating the transition to the proliferative phase. This aligns perfectly with our observed reduction in pro-inflammatory markers. Secondly, the robust promotion of cell proliferation and re-epithelialization strongly implies the involvement of the MAPK/ERK cascade. Literature firmly establishes that the PPIA-CD147 complex is a potent upstream activator of ERK1/2 signaling [31]. It is therefore a reasonable inference that GA-Gel, through upregulating PPIA, potentiates the MAPK/ERK pathway, thereby driving the proliferation and migration of fibroblasts and keratinocytes to accelerate tissue repair. Furthermore, the enhanced collagen deposition and improved tissue remodeling we documented are hallmarks of activated TGF-β signaling. PPIA has been identified as an enhancer of TGF-β signal transduction [33]. Consequently, we postulate that PPIA serves as the critical link whereby GA-Gel amplifies the TGF-β1 pathway, promoting fibroblast differentiation into myofibroblasts and subsequent extracellular matrix synthesis. In summary, we propose an integrative model wherein GA-Gel, via the central signaling node PPIA, acts as a molecular conductor to synergistically temper NF-κB-driven inflammation, propel MAPK/ERK-mediated proliferation, and augment TGF-β-dependent tissue remodeling. This multi-targeted action provides a compelling theoretical foundation for GA-Gel’s efficacy and underscores PPIA’s role as a key coordinator in the healing process, directly addressing the reviewer’s insightful comment.

## 4. Methods

### 4.1. The Target of SJYHO

Use the TCM Integrative Pharmacology Research Platform (TCMIP 2.0) at http://www.tcmip.cn (accessed on 11 August 2025), and select the herbs “Glycyrrhiza uralensis Fisch.”, “Angelica dahurica (Fisch. ex Hoffm.) Benth. & Hook. f. ex Franch. & Sav.”, “Angelica sinensis (Oliv.)Diels”, “Lithospermum erythrorhizon Siebold & Zucc.” and “Resina Draconis” to collect active components and relevant targets of SJYHO. Retrieve SMILES sequences from PubChem, and use SwissTargetPrediction (http://www.swisstargetprediction.ch (accessed on 11 August 2025)) by selecting “Homo sapiens” and “Probability > 0”. Obtain MOL2 component files from the Traditional Chinese Medicine System Pharmacology Database (TCMSP) at https://www.tcmsp-e.com/ (accessed on 11 August 2025) and use Pharmmapper (https://www.lilab-ecust.cn/pharmmapper/index.html (accessed on 11 August 2025)) to predict targets by selecting “Human protein targets only”. Convert results to “Gene Name” via the UniProt database at https://www.uniprot.org (accessed on 11 August 2025). Finally, integrate and deduplicate to obtain potential drug targets.

### 4.2. Targets Related to the Indications of SJYHO

Search GeneCards (https://www.genecards.org (accessed on 11 August 2025)) and CTD (https://ctdbase.org (accessed on 11 August 2025)) databases for targets related to Acne, Burns, Pressure ulcers, and Diabetic foot ulcers. Consolidate these targets to obtain disease-related ones. Use Wei Sheng Xin (https://www.bioinformatics.com.cn (accessed on 11 August 2025)) to find the intersection between disease targets and SJYHO targets, and visualize the drug–disease relationship with a radiation diagram.

### 4.3. Analysis of Wound-Related Targets

Search GEO (https://www.ncbi.nlm.nih.gov/geo/ (accessed on 11 August 2025)) for wound-related datasets. In Rstudio2025.05.01, use the limma package for data cleaning, apply normalizeBetweenArrays for standardization, identify differentially expressed genes with the Wilcoxon rank-sum test, and control the FDR using Benjamini–Hochberg. Set |logFC| > 3 and FDR < 0.3 as the criteria. Present results via volcano plots and heatmaps.

### 4.4. Analysis of Immune Infiltration

Utilize the CIBERSORT algorithm in R to conduct immune cell infiltration analysis on genomic data. Use the LM22 signature matrix to deconvolve the data and calculate the abundance of 22 immune cell types. Visualize the infiltration levels by creating scatter plots, heatmaps, and stacked bar charts. Additionally, use Origin 2025 to draw violin plots for immune infiltration. Based on the CIBERSORT results, filter wound-healing samples, compute the median expression of immune cells, and plot a pie chart to show their overall expression in these samples.

### 4.5. Machine Learning

Intersect the potential targets of SJYHO with GEO-identified differentially expressed genes to obtain the intersected genes. Then, perform dimensionality reduction on these genes using three machine learning methods: SVM-RFE, random forest, and LASSO. SVM-RFE: Use a five-fold cross-validation framework to assess feature importance. Determine the optimal feature subset via feature-number scanning and plot error rate vs. accuracy. Random Forest: Build a classification model with the randomForest package. Generate a random forest plot and rank genes by importance. LASSO: Confirm the best regularization parameter λ using five-fold cross-validation. Plot the cross-validation results to show MSE and standard error for different λ values. Extract λ.min and λ.1se, fit the LASSO regression path, and plot the path.

### 4.6. Component Backtracking of Core Targets

Identify core genes by intersecting genes screened via the three machine learning methods. Trace these core genes back to drug components. Integrate and classify the components hierarchically by TCM categories and compound types. Import the data into Wei Sheng Xin (https://www.bioinformatics.com.cn (accessed on 15 August 2025)) to create a pie chart showing the proportion of drug components. Select the most critical potential components for the experiment.

### 4.7. Preparation of Glycyrrhizic Acid-Containing Composite Gel

Glycyrrhizic acid was purchased from Chengdu Push Bio-Technology Co., Ltd., Chengdu, China (catalog no. PU0054-0025). Sodium diclofenac gel was obtained from Shenyang Luzhou Pharmaceutical Co., Ltd., Shenyang, China (catalog no. H19990010). SJYHO was from Huangshan Huibintang Biotechnology Co., Ltd., Huangshan, China (catalog no. Wan GZ WZ 2015000527).

Accurately weigh carboxymethyl cellulose, xanthan gum, and glycerol using a balance, moisten them by stirring, and gradually add distilled water while continuously stirring with a glass rod. Grind for about an hour to allow the gel to fully swell. Add triethanolamine and continue grinding, then add glycyrrhizic acid, sodium hyaluronate, 1,2-hexanediol, 1,2-octanediol, and 1,3-butanediol. Stir until a gel forms. Glycyrrhizic acid concentration, determined by prior research, is divided into three groups: 5% (GA-Gel-L), 10% (GA-Gel-M), and 15% (GA-Gel-H). The blank glycyrrhizic acid (GA) group is made by mixing 8.5 mL distilled water with 1.5 g glycyrrhizic acid. The blank gel (Gel) group is the water gel without glycyrrhizic acid. After the preparation is completed, let the gel stand out of light to remove air bubbles. Among them, the dosage of carbomer, xanthan gum, compound humectant and triethanolamine was optimized through single-factor response surface experiments, and stability tests (properties, Ph, heat resistance, cold resistance, humectability, sensory evaluation, etc.) were passed to ensure the best performance of the gel agent.

### 4.8. Wound Repair Experiment of Mice

Male BALB/c mice (20.0 ± 2.0 g, 6 weeks old) were purchased from SPF (Beijing) Biotechnology Co., Ltd. (License No.: SCXK (Jing) 2019-0010). Mouse experiments were approved by the Animal Ethical and Welfare Committee of Shanxi University of Chinese Medicine (the permission number: AWE202503313), and the laws of animal experiments were strictly observed. Mice were housed in a controlled environment (25 ± 2 °C, 60 ± 5% humidity, 12 h light/dark cycle) with free access to food and water for a week.

Mice were randomly divided into seven groups: Control group, GA group, Gel group, SJYHO group, GA-Gel-L group, GA-Gel-M group, and GA-Gel-H group, with six mice per group. After ether anesthesia, depilate the back skin, punch a 15.0 mm full-thickness skin defect, and dress with sterile gauze. House mice individually. The Control group receives 0.5 g of distilled water, while the other groups receive 0.5 g of respective drugs. Administer drugs twice daily for 17 days. Monitor mice for hair, movement, and eating changes. Cover the wound with PU membrane, take photograph, draw the outline, and measure the wound area using ImageJ 1.54p. Calculate the healing rate: [(Original area-unhealed area)/Original area] × 100%. Use GraphPad Prism 10.4.1 to draw line charts and conduct two-way ANOVA.

### 4.9. Histopathological Examination

On day 17 post-surgery, collect the wounded skin, fix it in 4% paraformaldehyde, and embed it in paraffin. Slice the embedded samples into 5 μm thick sections, and stain them with Hematoxylin–Eosin (H&E), Masson’s trichrome, and Sirius Red (Sigma-Aldrich, St. Louis, MO, USA). Use H&E staining to evaluate granulation tissue formation and re-epithelialization, and quantify the granulation tissue and epithelium regeneration using ImageJ. Analyze collagen deposition and the ratio of types I and III collagen using Masson’s trichrome and Sirius Red staining.

To assess angiogenesis on day 17 post-surgery, perform immunofluorescence (IF) staining with CD31 (1:200, ab32457, Abcam, Waltham, MA, USA) and α-SMA (1:200, ab5831, Abcam, USA) antibodies. To evaluate the inflammatory response on day 17 post-surgery, conduct IF staining with CD68 (1:200, ab201340, Abcam, USA) and CD206 (1:200, Ab64693, Abcam, USA) antibodies for macrophages and M2-macrophages. Stain cell nuclei with DAPI.

Acquire IF and immunohistochemical images using a confocal microscope (Olympus FV10 inverted microscope, Tokyo, Japan) and an inverted microscope (Axio Vert A1, Carl Zeiss, Oberkochen, Germany). Quantify the results with ImageJ and plot bar charts using GraphPad Prism 10.4.1. Perform one-way ANOVA to analyze significant differences.

### 4.10. Analgesia Experiment

Place mice on a heat plate preheated to 55 °C (±0.5 °C) at 25 °C room temperature. The time from contact with the hot plate to licking the hind paw was used as the pain threshold. Select mice with thresholds of 5–30 s. Measure pre-drug thresholds, then post-drug thresholds. Record the data, import it into GraphPad Prism 10.4.1, plot bar charts, and conduct one-way ANOVA for significant difference analysis.

### 4.11. Correlation Analysis

To clarify the role of immune cells in the sample, the CIBERSORT deconvolution algorithm was used to calculate the relative abundance of 22 immune cells. Then, the Spearman rank-correlation coefficient matrix between immune cell abundance and gene expression was calculated. The correlation between core genes and immune cells was visualized using a heatmap. The pROC package in R was used to plot the ROC curve and calculate the area under the curve (AUC) value.

### 4.12. Molecular Docking

First, obtain the sequence of the PPIA protein from the UniProt database. Then, use the sequence name to download the crystal structure from the PDB database (https://www.rcsb.org (accessed on 25 August 2025)). Retrieve the three-dimensional structure of glycyrrhizic acid from the PubChem database. Optimize the protein receptor and small-molecule ligand through a stepwise pre-processing procedure. Use AutoDockTools 1.5.7 to remove water molecules from the target protein and add hydrogen atoms, saving it as the receptor file. Import the small molecule into OpenBabel version3.1.1 and convert it to PDB format. Then, use AutoDockTools to add hydrogen atoms, assign molecular charges, and set the grid parameters for the docking area, generating a Vina-dedicated configuration file. Perform 10 independent docking calculations using AutoDock Vina to explore the best binding mode between the ligand and receptor. Screen for the lowest binding energy, retain the protein conformation with the optimal binding energy, and save it in PDB format. Visualize the best binding state of the ligand and receptor using PyMol version3.1.4.1 and analyze the ligand–receptor interaction pattern.

### 4.13. Molecular Dynamic

Use AlphaFold to remodel the protein receptor. Use PyMol to restore the protein position and convert it to standard PDB format. Complete the hydrogen atoms of the small molecule and save it as mol2 format. Generate molecular topology files using Sobtop1.0 software. Build the simulation system with GROMACS2020.6: create a water box model, parameterize with the AMBER99SB force field, solvate the system using the SPC water model, and neutralize the system by adding ions. Perform energy minimization using the steepest descent and conjugate gradient methods for 10,000 steps each. In the NVT and NPT equilibration stages, use the velocity Verlet integrator. Couple the temperature with V-rescale and the pressure with C-rescale. Each stage lasts for 2 ns. For the molecular dynamic simulation, set a time step of 2 fs for a total of 200 ns. Record trajectory frames every 10 ps to obtain 20,000 conformations. Maintain a constant temperature of 300 K. Apply a positional restraint of 1000 kJ/(mol·nm^2^) to the ligand. Analyze the structure using GROMACS commands to calculate Root mean square deviation (RMSD), Root mean square fluctuation (RMSF), Radius of gyration (Rg), Solvent accessible surface area (SASA), and Hydrogen bonds (Hbond). Visualize the xvg data files with qtGrace0.2.6.

### 4.14. Reverse Transcriptase Quantitative Polymerase Chain Reaction (RT-qPCR)

RT-qPCR was used to quantify PPIA expression. cDNA was synthesized from RNA using the SuperScript IV Reverse Transcription Kit. The cDNA was diluted to 1 ng/µL. All primer efficiencies were between 90 and 110%, ensuring reliable quantification. The primers were used as follows: PPIA (5′-AGCATACAGGTCCTGGCATCTTGT-3′, 5′-CAAAGACCACATGCTTGCCATCCA-3′), and GAPDH (5′-CGACTTCAACAGCAACTCCCACTCTTCC-3′, 5′-TGGGTGGTCCAGGGTTTCTTACTCCTT-3′). The PCR was performed on a C1000™ Thermal Cycler (Bio-Rad, CA, USA) with these steps: 1 cycle of 95 °C for 30 s, then 40 cycles of 95 °C for 5 s, the annealing temperature for 30 s, and a melting curve. Data were normalized to GAPDH using the ΔΔCt method.

### 4.15. Statistical Analysis

One-way ANOVA and multiple comparisons were executed using GraphPad Prism. Results are expressed as mean ± SEM to reflect data variability. Intergroup differences were evaluated with a t-test, where *p* < 0.05 was deemed statistically significant.

## 5. Conclusions

In conclusion, PPIA and glycyrrhizic acid are key for wound healing effects of SJYHO. Glycyrrhizic acid-containing composite gel can accelerate wound healing by inhibit late-stage angiogenesis, reducing inflammation, alleviating pain, and enhancing collagen deposition. And it exerts these beneficial effects by targeting PPIA, which is likely involved in mediating wound immune infiltration by regulating CD8^+^ T cells, neutrophils, and memory B cells. This includes strong molecular docking affinity and stable binding dynamics between glycyrrhizic acid and PPIA. This will provide more effective treatment strategies for clinical treatment of various wounds.

## Data Availability

The data presented in this study are available on request from the corresponding author due to privacy and ethical restrictions.

## Abbreviations

AUC, Area under the curve; GA-Gel, Glycyrrhizic acid-contained composite gel; Hydrogen bonds, Hbond; H&E, Hematoxylin-Eosin; IF, Immunofluorescence; RT-qPCR, Reverse transcriptase quantitative polymerase chain reaction; Rg, Radius of gyration; RMSD, Root mean square deviation; RMSF, Root mean square fluctuation; SJYHO, Shengji Yuhong Ointment; SASA, Solvent Accessible Surface Area; TCMIP, Traditional Chinese medicine integrative pharmacology research platform; TCMSP, Traditional Chinese medicine systems pharmacology database; pTM, Predicted template modeling score.
